# Partial rupture of the quadriceps muscle in a child

**DOI:** 10.1186/1471-2474-11-214

**Published:** 2010-09-19

**Authors:** Gokhan Aydemir, Selami Cakmak, Secil Aydinoz

**Affiliations:** 1 Department of Pediatrics, Aksaz Military Hospital 48150, Marmaris, Mugla, Turkey; 2 Department of Orthopaedic Surgery, Aksaz Military Hospital48150, Marmaris, Mugla, Turkey; 3 Department of Pediatrics, GATA Haydarpasa Training Hospital, Istanbul, Turkey

## Abstract

**Background:**

The quadriceps femoris muscle ruptures usually occur in the middle-aged population. We present a 4-year-old patient with partial rupture of the quadriceps femoris muscle. To our knowledge, this is the youngest patient reported with a quadriceps femoris muscle rupture.

**Case Presentation:**

A 4-year-old girl admitted to our clinic with left knee pain and limitation in knee movements. Her father reported that she felt pain while jumping on sofa. There was no direct trauma to thigh or knee. We located a palpable soft tissue swelling at distal anterolateral side of thigh. The history revealed that 10 days ago the patient was treated for upper tract respiratory infection with intramuscular Clindamycin for 7 days. When we consulted the patient with her previous doctor and nurse, we learnt that multiple daily injections might be injected to same side of left thigh. MRI showed a partial tear of vastus lateralis muscle matching with the injection sites. The patient treated with long leg half-casting for three weeks. Clinical examination and knee flexion had good results with conservative treatment.

**Conclusions:**

Multiple intramuscular injections may contribute to damage muscles and make prone to tears with muscle contractions. Doctors and nurses must be cautious to inject from different parts of both thighs.

## Background

Quadriceps muscle tears usually seen in middle-aged and older people[1-3]. Particularly people with chronic diseases (e.g. diabetes mellitus, renal failure, and gout) are prone to quadriceps muscle ruptures [4,5]. Ruptures of quadriceps muscle are rare in children and limited cases were reported in literature [4,6-8]. We report a partial rupture of quadriceps muscle (vastus lateralis part) in a 4-year-old girl after multiple intramuscular antibiotic injections.

## Case Report

**Figure 1:**
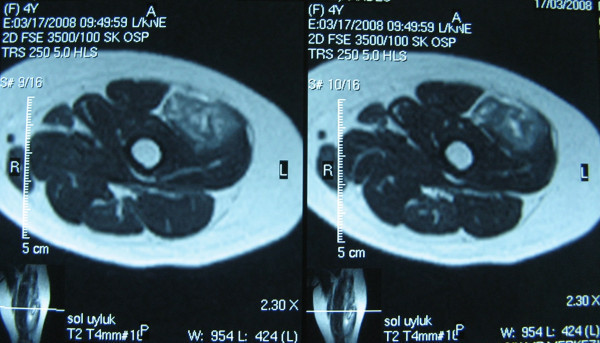
**Axial MR images show tear in vastus lateralis muscle**.

**Figure 2:**
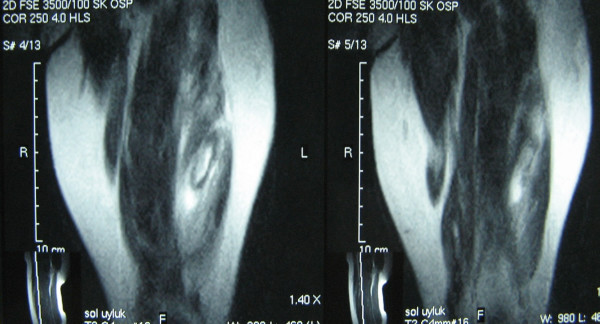
**Coronal MR images show hemorrhagic and rupture sites**.

A 4-year-old girl admitted to our clinic with left knee pain and limitation in knee flexion. She was holding her left leg at full extension. Her father said that she felt pain and fell down while she jumping on sofa. There was no trauma history. Physical examination revealed a localized palpable soft tissue swelling at anterolateral side of distal left thigh. Knee flexion was restricted. In detailed history we learnt that she had a serious upper tract respiratory infection and used some parenteral antibiotics (twice a day, intramuscular Clindamycin for 7 days). Intramuscular injections were applied to both thighs and ceased 10 days ago. We consultated patient with nurses and we learnt that multipl daily injections might be injected to the same area of left thigh. Plain radiographs revealed nothing. MRI showed a partial tear of vastus lateralis muscle matching with the injection site (Figures 1, 2). The patient placed in a long leg half-casting for three weeks. After this period, casting was taken off. The patient was symptom-free with full range of knee motion.

## Discussion

Quadriceps muscle tears are not common in children. These injuries were mainly described in middle-aged and older people. With aging and systemic illnesses (e.g. diabetes mellitus, renal diseases, obesity, gout, and rheumatoid arthritis), degeneration and weakness may occur in muscles and tendons. In adults, the weakest area in muscle-tendon-bone structural unit is myotendinous junction regarding tear mechanism. But in children the weakest point is physis. In a healthy child an avulsion fracture is more likely than a tendon rupture.

Possible complications of intramuscular injections include fibrosis and contracture [9]. Quadriceps muscle fibrosis and degeneration of muscle fibers can develop after multiple intramuscular injections. Disorganization of collagen fibers at these injection sites and weakening of muscle fibers can produce ruptures after muscle contractions and isotonic movements. The contents of substance injected inside the muscle can cause some kind of reactions and tissue response. This reaction is associated with muscle degeneration and inflammation [9]. The number of injections is also important for muscle fiber damage. Multiple injections into the same area may increase the risk of complications.

Diagnostic ultrasonography and MRI can be quite useful to confirm the possible diagnosis. In partial tears MRI may also be useful to determine the extent of injury.

## Conclusions

There are few reports in literature about quadriceps rupture in children and adolescents, but our case is the youngest patient in literature [4,6-8] And also there are no previous history excluding multiple intramuscular injections. With injection into the same muscle area multiple times, muscle may weaken and predispose to tears by muscle contractions. We think that nurses and other health officers must be careful about intramuscular injection sites and avoid from injecting repeatedly to the same areas. On the other hand, because of vastus lateralis has a serious role in stabilization of femoropatellar articulation, injections in this muscle must be lessen. MRI would be a good choice for exact diagnosis of such injuries in orthopedic and pediatric clinics.

## Competing interests

The authors declare that they have no competing interests.

## Authors' contributions

Our contributions are notified in the conclusions paragraph. GA experienced the case and participated in the design of the study. SC consulted the patient from orthopaedic view, and ordered MRI for diagnosis. SA collected the findings and drafted the manuscript. All authors read and approved the final manuscript.

## Pre-publication history

The pre-publication history for this paper can be accessed here:

http://www.biomedcentral.com/1471-2474/11/214/prepub
